# Effects of Pilates on Pain, Physical Function, Sleep Quality, and Psychological Factors in Young Women with Dysmenorrhea: A Preliminary Randomized Controlled Study

**DOI:** 10.3390/healthcare11142076

**Published:** 2023-07-20

**Authors:** Bo-Hwa Song, Jaehee Kim

**Affiliations:** Graduate School of Alternative Medicine, Kyonggi University (Seoul Campus), 24, Kyonggidae-ro 9-gil, Seodaemun-gu, Seoul 03746, Republic of Korea; sg060337@naver.com

**Keywords:** dysmenorrhea, premenstrual syndrome, Pilates, muscle strength, flexibility, sleep quality, psychological factors

## Abstract

The effect of Pilates on dysmenorrhea has been little studied. The purpose of this study was to evaluate the effect of Pilates on menstrual pain and symptoms, premenstrual syndrome, and risk factors of dysmenorrhea. Thirty young women with primary dysmenorrhea were randomly assigned into a Pilates group (PG; *n* = 15) and a waitlist control group (CG; *n* = 15). The Pilates was performed twice a week for 12 weeks. Menstrual pain and symptoms were measured by visual analogue scale (VAS) and the Cox menstrual symptom scale (CMSS), respectively. Premenstrual syndrome was assessed using the premenstrual symptoms screening tool (PSST). Additionally, back flexibility, hip muscle strength, sleep duration and quality, perceived stress, state-trait anxiety, and depression were evaluated. The VAS, CMSS severity and frequency, and PSST symptoms and functional impairments decreased in the PG compared to the CG (*p* < 0.001 or *p* < 0.01) with large effect sizes. Back flexibility and the strength of hip flexors, hip extensors, and hip abductors significantly increased in the PG compared to the CG (all *p* < 0.01) with large effect sizes. Sleep quality (*p* < 0.01) and stress (*p* < 0.05) improved in the PG. Sleep duration, anxiety, and depression did not change in either group. In conclusion, the 12-week Pilates intervention ameliorates dysmenorrhea, partly mediated by improved physical function and sleep quality.

## 1. Introduction

Dysmenorrhea, characterized by severe cramping pain in the lower abdomen during menstruation, negatively affects women’s quality of life and socioeconomic activities [1]. Dysmenorrhea can be classified as primary, with the absence of pelvic pathology, or secondary [2]. Secondary dysmenorrhea is mainly caused by endometriosis, uterine fibroids, and pelvic inflammatory disease [2]. Primary dysmenorrhea is more common and occurs predominantly in adolescents and young women [1,3]. Primary dysmenorrhea has a multifactorial etiology [2] and various risk factors, including biological factors (i.e., family history of dysmenorrhea, early menarche, and premenstrual syndrome), lack of physical activity, sleep disturbance, lumbopelvic misalignment, and small amount of abdomen muscles [1,3,4]. Recently, it has also been reported that dysmenorrhea is related to psychological factors, such as stress, depression, and anxiety [3,5].

Although the etiology of primary dysmenorrhea is not clear, uterine muscle ischemia, which occurs due to excessive muscle contraction during menstruation, is considered a pathophysiological cause of primary dysmenorrhea [3,6]. Decreased progesterone hormone in the luteal phase and overproduction of prostaglandins and inflammatory factors are considered to result in ischemia and hypoxia of the uterus, leading to increased perception of pain in dysmenorrhea [6,7,8,9]. Given this, a treatment approach that alleviates these pathophysiological factors and targets the known risk factors has been proposed as a plausible therapeutic approach to relieve primary dysmenorrhea [10].

Pharmacological therapies, such as non-steroidal anti-inflammatory drugs and oral contraceptives, are used to treat primary dysmenorrhea [2,11]. However, some individuals do not respond to these drugs, and the effectiveness of oral contraceptives still requires further research [2,11]. Therefore, various non-pharmacological therapies are utilized, and one that has been proven to be effective is exercise therapy [12,13]. Recent meta-analysis studies have reported that exercises, such as aerobic exercise, stretching, and muscle strengthening exercises, alleviate the severity of menstrual pain [9,14]. However, more research is needed as the specific type and amount of exercise have not yet been well established [9,14].

The number of people practicing Pilates is steadily increasing [15]. Pilates is proposed as a mind–body exercise that focuses on breathing and achieving proper muscle strength, flexibility, trunk stability, and posture [16]. In the last 10 years, most of the clinical studies on the effects of Pilates have been conducted to examine its pain relief effects, and it has been reported that Pilates relieves various types of pain, such as back pain and neck pain [17,18,19,20]. However, there has been relatively little research conducted on the effect of Pilates on dysmenorrhea [21,22].

In addition, the therapeutic mechanisms of Pilates for dysmenorrhea are currently unknown. Interestingly, prostaglandins have been reported to mediate the effect of Pilates on dysmenorrhea [21,22]. Following Pilates intervention, a decrease in prostaglandin levels, which cause hyperactivity of uterine muscles and menstrual pain, has been observed along with the relief of menstrual pain and symptoms in adolescents and young women [21,22]. Pilates has been proven to increase core strength, abdominal muscle mass, flexibility, and correct spinal alignment [14,23,24,25]. The improvement of core muscle strength and flexibility is often accompanied by the relief of back pain [26,27]. Similarly, it is plausible that Pilates could affect the physiology and function of lumbopelvic muscles, thereby resulting in the alleviation of menstrual pain and symptoms.

Sleep quality, stress, anxiety, and depression are known to be associated with dysmenorrhea [1,3,5]. Although their causal effects on dysmenorrhea are unknown, these psychological factors and dysmenorrhea could affect each other, leading to a vicious circle [28]. It is plausible that the effects of Pilates on dysmenorrhea may occur, in part, by affecting sleep quality and psychological factors. However, this has not been studied. A recent study reported that 12 weeks of Pilates training reduced depression and anxiety and improved sleep quality in postmenopausal women [29].

Therefore, in this study, our aim was to investigate whether Pilates relieves menstrual pain and symptoms and premenstrual syndrome in young women, in order to determine whether Pilates is an appropriate intervention method for alleviating dysmenorrhea. Additionally, this feasibility study aimed to examine how Pilates affects dysmenorrhea by evaluating its impact on the strength and flexibility of hip muscles, sleep quality, and psychological risk factors, including anxiety, depression, and stress.

## 2. Materials and Methods

### 2.1. Study Design and Procedures

**Figure 1:**
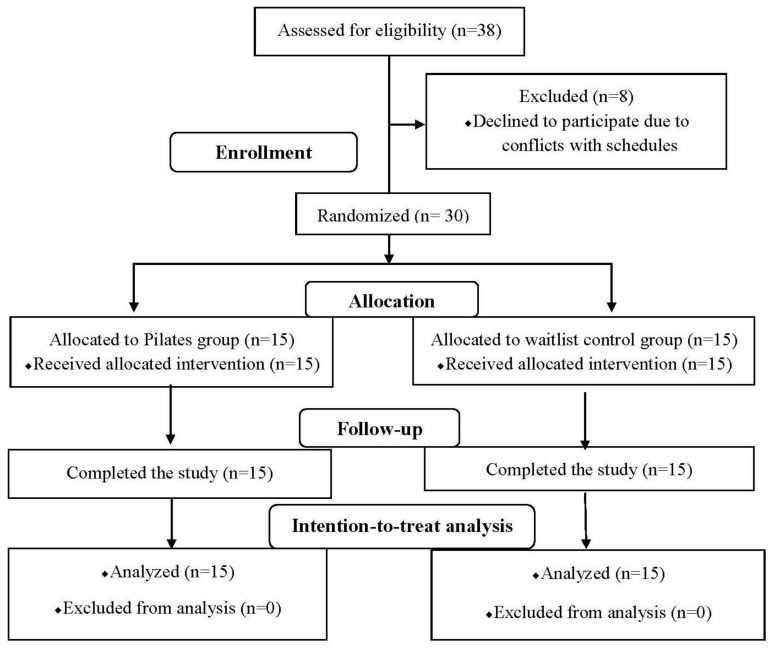
The study flowchart.

The study was a randomized controlled trial conducted from April 2021 to March 2022. The study complied with the guidelines of the Consolidated Standards of Reporting Trials (CONSORT) [30]. Recruitment was conducted by posting a research flyer on various social network service platforms (https://www.daangn.com/ (accessed on 1 May 2021), Instagram, Facebook, University community) in Seoul, Republic of Korea. A total of 38 participants were screened for eligibility based on inclusion and exclusion criteria. The CONSORT flowchart of the study is presented in Figure 1. Thirty subjects were enrolled in the study and randomly assigned to either the Pilates experimental group (*n* = 15) or the waitlist control group (*n* = 15) using a lottery method for sample randomization. In the order of study enrollment, subjects chose a sealed and opaque envelope from a box containing ten envelopes. Their group assignment was determined by the labeled paper inside the envelope, indicating either number 1 (Pilates group) or number 2 (control group). The box contained ten envelopes, with five labeled as number 1 and five labeled as number 2.

The Pilates group underwent a total of 24 Pilates sessions over a period of 12 weeks. The waitlist control group did not receive any treatment during the 12-week period but later participated in the same Pilates program as the Pilates group. During the study, this control group was used as an untreated comparison group and subsequently underwent the same treatment after the study concluded. All randomized subjects completed the study. Finally, data from 15 subjects in the Pilates group and 15 subjects in the waitlist control group were analyzed based on an intention-to-treat analysis, which included all randomized subjects in the analysis [31].

Measurements of menstrual pain and symptoms, symptoms of premenstrual syndrome, back flexibility, hip muscle strength, sleep duration and quality, and psychological factors, including perceived stress, depression, and anxiety, were taken at baseline and after 12 weeks.

### 2.2. Ethical Considerations

Ethical approval for this study was obtained from the Institutional Review Board of Kyonggi University (KGU-20201119-HR-062-04). All participants provided written informed consent before participating in the study. The study was registered with the Korean Clinical Trial Registry (CRIS.nih.go.kr: KCT0006903), which is accessible through the WHO International Clinical Trials Registry Platform (ICTRP).

### 2.3. Subjects

The inclusion criteria for study participation were young women aged 19–39 years with primary dysmenorrhea. The exclusion criteria included pregnancy, infectious disease, neurological disease, taking psychological medications, gynecologic diseases including endometriosis, pelvic inflammatory disease, uterine cancer, or receiving non-pharmacological treatment for dysmenorrhea.

### 2.4. Pilates Protocol

**Figure 2:**
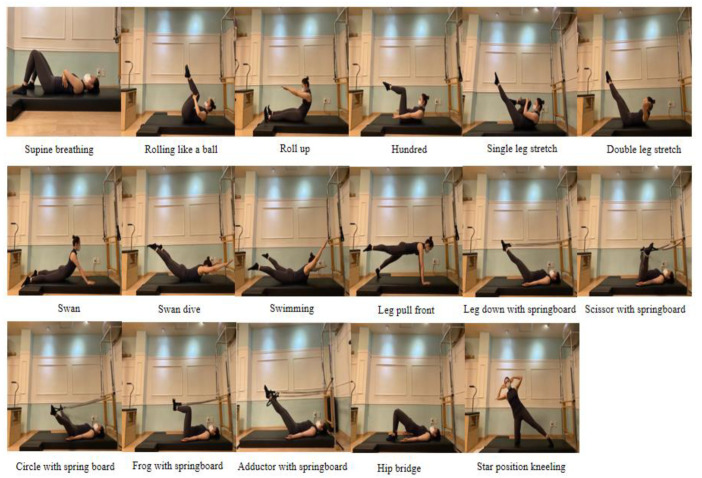
The mat Pilates program.

The Pilates program was conducted at a private Pilates center in Seoul, twice a week at 8 PM, with each session lasting 50 min. The Pilates exercises were performed as group exercise classes with participants wearing face masks, following the guidelines necessitated by the COVID-19 pandemic. Each class consisted of 5 subjects. The program consisted of a warm-up exercise and a series of mat Pilates exercises [20]. The warm-up exercise included 6 foam roller stretches, which consisted of head tilt, supine back rolling for the upper and lower back and sacrum, calf stretch, and serratus anterior stretch. The series of 17 mat Pilates exercises, designed to relax and strengthen the back, abdominal, and pelvic muscles, were performed as presented in Figure 2 [32,33].

The Pilates exercises were divided into 2 programs. The 1st program was performed every Tuesday, and the 2nd program was performed every Friday for 12 weeks. Both programs started with supine breathing, rolling like a ball, and roll up and finished with roll up and supine breathing. Additionally, the 1st program included hundred, single leg stretch, double leg stretch, swan, swan dive, swimming, leg pull front, and star position kneeling. The 2nd program included hundred, single leg stretch, double leg stretch, leg down with springboard, scissor with springboard, circle with springboard, frog with springboard, and hip bridge. Each exercise in the 1st and 2nd programs was done with 3 sets of 10 repetitions and with 10–15 s of rest between sets. The repetition was progressively increased up to 15. All Pilates exercise sessions were led by one instructor who had 8 years of experience in teaching Pilates.

### 2.5. General, Menstrual, and Lifestyle Characteristics

At baseline, general, menstrual, and lifestyle characteristics of the subjects were assessed (Table 1). Age, marital status, height, and weight were measured. Body mass index (BMI) was calculated by dividing the weight in kilograms by the square of the height in meters. The percentage of body fat was measured using bioelectrical impedance analysis (InBody230, InBody Co., Ltd., Seoul, Republic of Korea). The body fat and BMI were retested after 12 weeks. Menstrual characteristic included age at menarche, family history of dysmenorrhea, length of menstrual cycle, volume of menstrual fluid, period length, and regularity. Lifestyle characteristics included smoking status (current smoker or non-smoker), alcohol consumption (current drinker or non-drinker), and exercise. Additionally, the medication, including painkillers for dysmenorrhea that subjects were taking was reported. Subjects were also asked to record the amount and frequency of pain medicine consumed during each menstruation.

### 2.6. Primary Outcomes

#### 2.6.1. Dysmenorrhea and Premenstrual Syndrome

The intensity of menstrual pain was measured by a visual analog scale (VAS), which is the most used measure of dysmenorrhea [10]. The VAS consists of a 10 cm horizontal line without markings, with the left and right ends labeled as ‘no pain’ and ‘very severe pain’, respectively [34]. The distance from the ‘no pain’ point was then measured in centimeters.

The frequency and severity of menstrual symptoms were evaluated using the Korean version of the Cox menstrual symptom scale (CMSS) [35,36]. The CMSS assesses the frequency and severity of 18 physical and emotional menstrual symptoms associated with dysmenorrhea [34]. The scale for each item ranges from 0 (not noticeable) to 4 (very severely bothersome) for the severity domain, and from 0 (did not occur) to 4 (lasted several days) for the frequency domain [34]. Total scores for the frequency and severity domains were calculated by summing up the scales of the 18 symptoms.

Symptoms of premenstrual syndrome (PMS) were assessed using the Korean version of the premenstrual symptoms screening tool (PSST) [37,38]. The PSST consists of 14 psychological, physical, and behavioral PMS symptoms, along with 5 measures of interference with daily activities [39]. The scale for each item ranges from 0 (not at all) to 3 (severe) [37]. The Korean version of the PSST has exhibited good internal consistency and reliability [40]. The total scores of the 14 symptoms and 5 functional impairments were calculated separately.

#### 2.6.2. Muscle Strength and Flexibility

The maximal isometric strength of the hip flexors, hip extensors, and hip abductors in both legs was assessed using a hand-held dynamometer, the MicroFET2 device (Hoggan Indiustries, Inc., West Jordan, UT, USA), as previously described [41,42]. The MicroFET2 device has been proven to be reliable for assessing the strength of these muscles [43].

The flexibility of the lower back and hamstrings was assessed using the sit-and-reach box (Sit & Reach Box, Lafayette Instrument Company, Lafayette, IN, USA). The sit-and-reach test has strong evidence supporting its high reliability [44].

### 2.7. Secondary Outcomes

#### 2.7.1. Sleep Quality and Duration

Sleep quality was assessed using the Pittsburgh sleep quality index (PSQI), which evaluates subjective sleep quality over the past month [45,46]. The PSQI consists of 19 items on a scale of 0–3 [45,46]. The Korean version of PSQI has been found to be valid and reliable [46,47]. Seven component scores, ranging from 0 (no difficulty) to 3 (severe difficulty), were derived and summed to produce a total score (ranging from 0 to 21) [45]. A total score greater than 5 indicates poor quality sleep [46]. Sleep duration was measured using the following item from the PSQI: “During the past month, how many hours of actual sleep did you get at night?” [45].

#### 2.7.2. Psychological Variables

The subjective stress level was evaluated using the Korean version of the 10-item Perceived Stress Scale (PSS) [48]. The total score of the PSS ranges from 0 to 40, with a higher score indicating greater levels of stress [49]. The validity and reliability of the Korean PSS have been well established [48].

Depression was assessed using the Korean version of the Center for Epidemiologic Studies Depression (CES-D) scale. Participants were asked to rate how they felt about 20 depressive symptoms during the past week on a scale of 0 (less than 1 day) to 3 (5–7 days) [50,51]. The Korean version of the CES-D has demonstrated good validity and high internal consistency [52]. A total score was calculated by summing the scales of the 20 items.

Anxiety was evaluated using the Korean version of the State-Trait Anxiety Inventory (STAI) form Y [53]. The STAI consists of two subtests, one assessing the current state of anxiety (state anxiety) and the other measuring anxiety proneness (trait anxiety) [54]. Each subtest has 20 items, with a total score ranging from 20 to 80, and a higher score indicates greater anxiety [54]. The reliability and validity of the Korean version of the STAI have been well established [53].

### 2.8. Sample Size

The sample size calculation was based on the minimal clinically important difference (MCID) for dysmenorrhea, measured by VAS (4 cm) [55]. Using the online Sample Size Calculator (https://homepage.univie.ac.at/robin.ristl/samplesize.php (accessed on 20 January 2021)), we determined the sample size of 15 for each group to detect the MCID of 4 cm, assuming a standard deviation of 1.25 (5 cm) with 80% power and a two-sided α = 0.05, when testing the significance of change from baseline using a paired t-test.

### 2.9. Statistical Methods

The data were analyzed using SPSS version 27.0 (IBM SPSS Statistics, Armonk, NY, USA). A significance level of 0.05 was set for all analyses. The normality of distribution for continuous numerical data was assessed using the Shapiro–Wilk test. The significance of the pre–post change within each group was tested using the paired t-test or Wilcoxon signed ranks test, depending on the normality of the distribution. To test the significance of group differences in baseline variables and pre–post changes, independent t-tests or Mann–Whitney U tests were performed. For categorical variables, the significance of group differences was assessed using the chi-square test and Fisher’s exact test.

Additionally, effect size estimates were calculated as follows: Cohen’s d for *t*-tests and r value for the Wilcoxon signed ranks test and Mann–Whitney test [56,57]. Cohen’s d was calculated using SPSS and was interpreted as small (d = 0.2), medium (d = 0.5), and large (d ≥ 0.8) effect size [56,58]. The r value was calculated as the Z statistic divided by the square root of the total sample size or the total number of pairs [57] and was interpreted as a small (r = 0.1), medium (r = 0.3), and large (r ≥ 0.5) effect size [58,59].

## 3. Results

### 3.1. Subject Characteristics

All 15 subjects in the Pilates group completed the entire intervention, consisting of 24 sessions. The subject characteristics of the Pilates and control groups are presented in Table 1. There were no significant group differences in age, BMI, body fat, marital status, and menstrual and lifestyle characteristics at baseline.

**Table 1:** Subject baseline characteristics.

Variables	Categories	Pilates Group (*n* = 15)	Control Group (*n* = 15)	*p*-Value ^a^
Age (years)		33.9 ± 3.5	31.3 ± 4.5	0.096
Body mass index (kg/m^2^)		21.4 ± 2.1	21.2 ± 1.9	0.818
Body fat (%)		31.0 ± 4.7	29.8 ± 5.1	0.504
Menarcheal age (year)		12.9 ± 1.1	12.9 ± 1.6	1.000
Period length (day)		5.3 ± 1.3	5.1 ± 1.2	0.683 ^b^
Length of menstrual cycle (day)		30.3 ± 5.1	29.9 ± 5.5	0.806 ^b^
Period regularity	Regular	14 (93.3)	7 (46.7)	0.014
	Irregular	1 (6.7)	8 (53.3)	
Volume of menstrual fluid	Light	3 (20.0)	0 (0.0)	0.117 ^c^
	Moderate	10 (66.7)	10 (66.7)	
	Heavy	2 (13.3)	5 (33.3)	
Family history of dysmenorrhea	No	2 (13.3)	5 (33.3)	0.135 ^c^
	Yes	8 (53.3)	9 (60.0)	
	Not know	5 (33.3)	1 (6.7)	
Marital status	Married	2 (13.3)	2 (13.3)	1.000
	Unmarried	13 (86.7)	13 (86.7)	
Smoking	No	15 (100.0)	14 (93.3)	1.000
	Yes	0 (0.0)	1 (6.7)	
Drinking	No	4 (26.7)	4 (26.7)	1.000
	Yes	11 (73.3)	11 (73.3)	
Moderate-intensity exercise (≥150 min/wk)	No	14 (93.3)	15 (100.0)	1.000
	Yes	1 (6.7)	0 (0.0)	
Sleep quality	Poor	15 (100.0)	12 (80.0)	0.224
	Good	0 (0.0)	3 (20.0)	
Use of pain relievers to manage dysmenorrhea	No	4 (26.7)	1 (6.7)	0.330
	Yes	11 (73.3)	14 (93.3)	

Data are presented as mean ± standard deviation or *n* (%). ^a^ Independent *t*-test for continuous variables or Fisher’s exact test for categorical variables. ^b^ Mann–Whitney U test. ^c^ Chi-square test.

Eleven subjects in the Pilates group (73.3%) and fourteen subjects in the control group (93.3%) had used pain medication for painful menstrual periods at baseline. Other medications taken by the subjects included levothyroxine for hypothyroidism (*n* = 1) and anti-inflammatory medicine for shoulder impingement syndrome (*n* = 1) in the Pilates group. Two subjects in the Pilates group had asthma and allergic rhinitis.

Additionally, at baseline, there were no significant group differences in all primary and secondary outcomes except VAS (*p* = 0.026), CMSS frequency (*p* = 0.021), PSST symptom (*p* = 0.010), PSST functional impairments (*p* = 0.009), sleep duration (*p* = 0.005), and depression (*p* = 0.045).

### 3.2. Changes in Primary Outcomes

As shown in Table 2, the paired t-tests and Wilcoxon signed rank tests revealed that in the Pilates group, VAS (*p* < 0.001), CMSS severity (*p* < 0.001) and frequency (*p* < 0.01), PSST symptoms (*p* < 0.001), and PSST functional impairments (*p* < 0.01) significantly decreased after the intervention with large effect sizes. Meanwhile, in the control group, no significant changes from baseline were observed in any of these variables. Independent t-tests showed that the pre–post changes in all menstrual variables were significantly larger in the Pilates group compared to the control group (all *p* < 0.001), with large effect sizes.

The results of the paired *t*-tests and Wilcoxon signed rank tests for flexibility and muscle strength are presented in Table 2. After the 12-week Pilates intervention, back flexibility significantly increased (*p* < 0.01), while it decreased in the control group (*p* < 0.05). The strength of hip flexors, hip extensors, and hip abductors in both sides significantly increased in the Pilates group (all *p* < 0.01), with large effect sizes. In the control group, the right hip flexor strength decreased after 12 weeks (*p* < 0.05), with a medium effect size, while other strength measures did not change. The results of the independent t-tests and Mann–Whitney U tests showed that the magnitude of changes in all measures was significantly greater in the Pilates group compared to the control group (all *p* < 0.01), with large effect sizes.

### 3.3. Secondary Outcomes

The results of secondary outcomes are presented in Table 3. The Wilcoxon signed rank test revealed that the PSQI total score significantly decreased in the Pilates group (*p* < 0.01) with a large effect size, but not in the control group. The Mann–Whitney U test showed that the group difference in the extent of the change was significant (*p* < 0.05) with a medium effect size. Sleep duration did not change in the Pilates group but decreased in the control group (*p* < 0.05) with a large effect size. However, the group difference in the change did not reach statistical significance.

Depression and state-trait anxiety did not change significantly in either group after 12 weeks, while PSS decreased only in the Pilates group (*p* < 0.05) with a medium effect size. However, the group differences in the extent of the PSS change were not significant.

Additionally, BMI did not change in either group over 12 weeks. Body fat percentage decreased in the Pilates group (*p* < 0.05), but the group difference in the extent of the change was not significant.

Qualitative analysis of the medication diary showed that seven subjects (46.7%) of the Pilates group did not take pain medicine during the intervention phase. The number of tablets and frequency of pain medicine consumed during menstruation had decreased in the remaining subjects in the Pilates group.

## 4. Discussion

The primary findings of this study were that the 12-week Pilates intervention alleviated menstrual pain intensity (VAS), menstrual symptoms (CMSS), and premenstrual symptoms (PSST). These improvements were observed concomitantly with enhanced physical function, including back flexibility and hip muscle strength. Importantly, the extent of improvement of VAS, CMSS, PSST, and physical function was significantly greater in the Pilates group compared to the no-treatment control group.

The MCID is a measure that reflects the meaningful improvement for patients and is often used to determine treatment effectiveness [60,61]. The MCID for menstrual pain has been reported to be 4 cm on the VAS scale after anesthetic treatment in patients with endometriosis [55]. In our study, following the Pilates intervention, the average decrease in VAS score was 5.2 cm, which exceeded the reported MCID. This suggests that the Pilates intervention has a clinically significant effect in relieving dysmenorrhea. However, the MCID for menstrual symptoms and PMS has not been reported.

In addition to menstrual pain, women with dysmenorrhea often experience various menstrual symptoms such as headache, nausea, vomiting, diarrhea, back pain, thigh pain, and insomnia [2,3]. It is known that PMS is associated with dysmenorrhea [1,3]. In our study, the Pilates intervention also resulted in a reduction in the severity and frequency of menstrual symptoms, severity of PMS symptoms, and functional impairment due to PMS.

A variety of non-pharmacological therapies, such as acupuncture, acupressure, transcutaneous electrical nerve stimulation, massage, heat therapy, and different types of exercises, have been applied for primary dysmenorrhea and have shown effectiveness in reducing menstrual pain intensity [2,9,10,12,13]. Interestingly, recent suggestions for complementary treatment options for dysmenorrhea and PMS include improving the mobility and motility of the uterus through manual visceral therapy [62].

Previous systematic reviews and meta-analyses on the effects of exercise on primary dysmenorrhea have shown that conventional exercises, including aerobic exercise, stretching, strength exercise, and yoga, can reduce the severity of menstrual pain [2,9,14]. Pilates, although not a traditional form of exercise or yoga, is defined as a mind–body exercise that promotes relaxation, mindfulness, and improvement of muscle strength, flexibility, core stability, and posture [16,63]. However, Pilates shares some characteristics with stretching, strength exercise, and yoga, such as the use of breathing techniques and yoga poses. In our study, after the 12-week Pilates intervention, there was a decrease in menstrual pain intensity. These findings are consistent with previous studies that have reported a reduction in menstrual pain intensity following conventional exercises and yoga [2,9,14]. Aerobic exercise and yoga have also been shown to be effective in improving PMS symptoms [64,65].

Limited research has been conducted regarding the impact of Pilates on primary dysmenorrhea and PMS. In our study, we observed a decrease in the severity of menstrual pain and symptoms following the Pilates intervention. These findings are consistent with previous studies that have reported a decrease in the severity of menstrual pain, as measured by the VAS and the McGill Pain Questionnaire, as well as a reduction in menstrual symptoms, as measured by the Menstrual Distress Questionnaire, after Pilates interventions [21,22,66,67]. In a recent study examining the effects of Pilates on PMS, Çitil and Kaya [68] reported that a 12-week Pilates exercise program improved PMS symptoms in college students.

The mechanisms underlying the effect of Pilates on dysmenorrhea are still unknown. It has been proposed that the improvement in physical function, which results from the relaxation and strengthening of target muscles, may play a role in pain relief, such as in the case of back pain [26,27]. In our study, we observed that after the Pilates intervention, there was an average increase of approximately 11–50% in isometric muscle strength of the hip flexors, extensors, and abductors, as measured by a hand dynamometer. This increase in muscle strength was accompanied by an improvement in dysmenorrhea. The test–retest measurement variation of hip strength assessment using the hand dynamometer has been reported to be below 5% for hip flexion and abduction and 8% for hip extension, suggesting that strength changes above 10% in the hip muscles represent real changes in healthy adults [43]. Additionally, we found that back flexibility increased by approximately 18% in the Pilates group. These findings provide preliminary evidence that strengthening the hip muscles may be involved in the beneficial effects of Pilates on dysmenorrhea.

Poor sleep quality has been linked to an increased risk of primary dysmenorrhea [69]. In our study, we observed a decrease in the PSQI total score following the Pilates intervention, indicating an improvement in sleep quality. However, sleep duration did not change significantly. Like our findings, a recent meta-analysis study reported that Pilates interventions resulted in reduced PSQI total scores in various populations, including postmenopausal women, middle-aged and elderly women, postpartum women, and hemodialysis patients [70]. Although not specific to dysmenorrhea, these findings support the potential benefits of Pilates on improving sleep quality across different populations.

Negative emotions such as anxiety, depression, and stress have been shown to have an impact on primary dysmenorrhea [5,69]. We hypothesized that the reduction in these negative emotions following the Pilates intervention may contribute to the improvement of dysmenorrhea and PMS. However, we did not observe any significant effects of the Pilates intervention on anxiety and depression. Although the stress level significantly decreased in the Pilates group, the extent of this change was not different from that observed in the control group. The effects of Pilates on mental stress in the context of dysmenorrhea are still not well understood. Therefore, further studies are needed to clarify the effects of Pilates on negative emotions in women with dysmenorrhea.

Many women with dysmenorrhea either tolerate the pain or use over-the-counter pain relievers [2]. In our study, we allowed the subjects to take pain medication if needed for dysmenorrhea during the intervention and asked them to record it. In the Pilates group, we observed a general decrease in the dosage and frequency of pain medication consumed for dysmenorrhea.

The study has some limitations. The lack of statistically significant intragroup differences in depression and anxiety could be attributed to the small sample size. Additionally, our results showed that the intergroup difference in stress change did not reach statistical significance. Therefore, further studies with a larger sample size will be needed to fully clarify the effects of Pilates on anxiety, depression, and stress in women with dysmenorrhea.

Another limitation of the study is the type of control group used. We compared the effect of Pilates on dysmenorrhea with that of a no-treatment control group. It is possible that the observed effects of Pilates could be influenced, at least in part, by placebo and/or socialization effects. Thus, further studies comparing Pilates with conventional exercise or pharmacological therapy for the management of dysmenorrhea will be necessary to better evaluate the effectiveness of Pilates.

## 5. Conclusions

In the current study, we demonstrated that a 12-week Pilates intervention in women with dysmenorrhea resulted in significant reductions in menstrual pain and symptoms, premenstrual symptoms, as well as improvements in back flexibility, hip muscle strength, and sleep quality when compared to no treatment. These findings suggest that Pilates may have beneficial effects on dysmenorrhea, potentially mediated by the improvement of hip muscle function and sleep quality. Collectively, Pilates might show promise as a complementary therapy for the management of dysmenorrhea.

## Figures and Tables

**Table 2:** Changes in primary outcomes.

Variables	Group	Baseline	Week 12	*p*-Value ^a^	Effect Size ^d^	Week 12—Baseline	*p*-Value ^c^	Effect Size ^d^
VAS (cm)	Pilates group	7.6 ± 1.2	2.4 ± 1.5	**0.000**	3.64	−5.2 ± 1.4	**0.000**	−3.30
	Control group	6.4 ± 1.4	6.7 ± 1.2	0.468 ^b^	0.19 ^e^	0.3 ± 1.9		
CMSS severity	Pilates group	30.1 ± 13.5	14.1 ± 9.7	**0.000**	1.40	−15.9 ± 11.4	**0.000**	1.75
	Control group	21.9 ± 9.3	21.7 ± 7.0	0.806 ^b^	−0.06 ^e^	0.2 ± 1.9		
CMSS frequency	Pilates group	34.0 ± 12.7	17.9 ± 12.5	**0.002** ^b^	−0.80 ^e^	−16.1 ± 14.0	**0.000**	−1.60
	Control group	24.3 ± 7.7	25.8 ± 7.4	0.411	−0.22	1.5 ± 6.7		
PSST symptoms	Pilates group	26.8 ± 9.7	14.7 ± 9.2	**0.000**	1.38	−12.1 ± 8.7	**0.000**	−1.92
	Control group	17.2 ± 9.5	20.5 ± 9.1	0.096	−0.46	3.3 ± 7.2		
PSST functional impairment	Pilates group	9.1 ± 3.4	4.5 ± 3.7	**0.001** ^b^	−0.89 ^e^	−4.6 ± 2.9	**0.000**	−1.83
	Control group	5.4 ± 3.2	6.4 ± 4.0	0.241	−0.32	1.0 ± 3.2		
Back flexibility (cm)	Pilates group	29.4 ± 6.8	33.5 ± 4.7	**0.006** ^b^	0.71 ^f^	4.1 ± 5.4	**0.000** ^d^	−0.70 ^f^
	Control group	31.8 ± 8.6	28.4 ± 8.2	**0.010**	0.77	−3.4 ± 4.5		
Muscle strength								
Left hip flexors (kg)	Pilates group	8.4 ± 0.9	11.8 ± 1.5	**0.000**	−1.81	3.5 ± 1.9	**0.000**	2.28
	Control group	8.2 ± 1.9	8.1 ± 1.3	0.764	0.08	−0.1 ± 1.1		
Right hip flexors (kg)	Pilates group	8.9 ± 1.7	11.7 ± 1.6	**0.001**	−1.13	2.8 ± 2.5	**0.000**	1.82
	Control group	8.3 ± 0.8	7.7 ± 0.9	**0.029**	0.63	−0.6 ± 1.0		
Left hip Extensors (kg)	Pilates group	10.3 ± 1.5	11.5 ± 1.4	**0.002**	−1.00	1.2 ± 1.2	**0.007**	1.06
	Control group	10.9 ± 2.0	10.7 ± 1.8	0.562	0.15	−0.2 ± 1.5		
Right hip Extensors (kg)	Pilates group	10.3 ± 1.7	12.0 ± 1.5	**0.000**	−1.46	1.6 ± 1.1	**0.000**	1.54
	Control group	11.2 ± 2.0	10.5 ± 1.9	0.168	0.38	−0.7 ± 1.8		
Left hip abductors (kg)	Pilates group	9.8 ± 1.4	12.3 ± 2.0	**0.002** ^b^	0.80 ^f^	2.4 ± 2.1	**0.000**	0.98
	Control group	9.7 ± 1.6	10.0 ± 1.9	0.619	−0.13	0.3 ± 2.2		
Right hip abductors (kg)	Pilates group	8.3 ± 1.6	12.4 ± 2.1	**0.000**	−1.46	4.1 ± 2.8	**0.004** ^d^	−0.51 ^f^
	Control group	9.5 ± 1.9	9.6 ± 1.7	0.844	−0.05	0.1 ± 2.1		

VAS: visual analogue scale, CMSS: Cox menstrual symptom scale, PSST: premenstrual symptoms screening tool. Data are presented as mean ± standard deviation. ^a^ The significance of the difference between baseline and week 12 in each group was tested by a paired t-test. ^b^ The significance of the difference between baseline and week 12 in each group was tested by the Wilcoxon signed ranks test. ^c^ The significance of the difference between the groups was tested using an independent *t*-test. ^d^ The significance of the difference between the groups was tested using the Mann–Whitney U test. ^e^ The Cohen’s d effect size. ^f^ The r effect size. The *p* values less than 0.05 are in bold.

**Table 3:** Changes in secondary outcomes.

Variables	Group	Baseline	Week 12	*p*-Value ^a^	Effect Size ^e^	Week 12—Baseline	*p*-Value ^c^	Effect Size ^e^
Body mass index (kg/m^2^)	Pilates group	21.4 ± 2.1	21.3 ± 1.7	0.531	0.17	−0.1 ± 0.6	0.129	−0.57
	Control group	21.2 ± 1.9	21.5 ± 2.0	0.148	−0.40	0.2 ± 0.6		
Body fat (%)	Pilates group	31.0 ± 4.7	29.9 ± 5.1	**0.018**	0.69	−1.1 ± 1.6	0.126 ^d^	0.28 ^f^
	Control group	29.8 ± 5.1	30.4 ± 5.7	0.347	−0.25	0.6 ± 2.5		
Total score of sleep quality	Pilates group	8.0 ± 2.0	6.0 ± 1.1	**0.006** ^b^	−0.71 ^f^	−2.0 ± 1.9	**0.015** ^d^	0.46 ^f^
	Control group	8.8 ± 3.5	8.6 ± 3.0	0.317 ^b^	−0.26 ^f^	−0.2 ± 0.8		
Sleep duration (hour)	Pilates group	6.3 ± 0.8	6.4 ± 1.1	0.675 ^b^	0.11 ^f^	0.1 ± 0.8	0.067 ^d^	−0.34 ^f^
	Control group	5.8 ± 1.1	4.9 ± 1.5	**0.040** ^b^	−0.52 ^f^	−0.9 ± 1.5		
Perceived stress	Pilates group	21.6 ± 3.5	20.0 ± 3.8	**0.036**	0.60	−1.6 ± 2.7	0.683 ^d^	0.12 ^f^
	Control group	20.7 ± 3.8	19.4 ± 4.6	0.195	0.35	−1.3 ± 3.8		
Depression	Pilates group	15.7 ± 8.9	12.0 ± 4.8	0.081	0.42	−3.7 ± 8.8	0.653 ^d^	0.08 ^f^
	Control group	21.1 ± 8.1	19.3 ± 10.6	0.267	0.30	−1.8 ± 6.0		
State anxiety	Pilates group	43.8 ± 9.1	40.1 ± 7.6	0.110	0.44	−3.7 ± 8.3	0.461 ^d^	0.14 ^f^
	Control group	43.8 ± 8.0	43.1 ± 11.0	0.729	0.09	−0.7 ± 8.0		
Trait anxiety	Pilates group	41.2 ± 6.7	39.6 ± 6.2	0.281	0.29	−1.6 ± 5.5	0.345 ^d^	0.18 ^f^
	Control group	45.5 ± 5.8	46.1 ± 7.7	0.864 ^b^	−0.10	0.6 ± 6.1		

Data are presented as mean ± standard deviation. ^a^ The significance of the difference between baseline and week 12 in each group was tested by a paired t-test. ^b^ The significance of the difference between baseline and week 12 in each group was tested by the Wilcoxon signed ranks test. ^c^ The significance of the difference between the groups was tested using an independent *t*-test. ^d^ The significance of the difference between the groups was tested using the Mann–Whitney U test. ^e^ The Cohen’s d effect size. ^f^ The r effect size. The *p* values less than 0.05 are in bold.

## Data Availability

The data presented in this study are available on request from the corresponding author.
